# 1114. Effectiveness and Safety of Beta-lactam Antibiotics with and without Therapeutic Drug Monitoring in Patients with *Pseudomonas aeruginosa* Pneumonia or Bloodstream Infection

**DOI:** 10.1093/ofid/ofab466.1308

**Published:** 2021-12-04

**Authors:** Ashlan J Kunz Coyne, Mohammad H Al-Shaer, Anthony M Casapao, Jason Ferreira, Carmen Isache, Christopher Jankowski

**Affiliations:** 1 UF Health Jacksonville, Jacksonville, Florida; 2 University of Florida, Gainesville, FL; 3 University of Florida College of Pharmacy, Jacksonville, Florida

## Abstract

**Background:**

*Pseudomonas aeruginosa* (PSAR) is challenging to treat due to its multiple resistance mechanisms, limited anti-PSAR agents, and population pharmacokinetic (PK) variances. Beta-lactam antibiotics (BLA) are commonly used to treat PSAR infections and although they have a wide therapeutic index, suboptimal exposures may lead to treatment failure and antimicrobial resistance while high exposure may result in adverse effects. Certain patient populations may benefit from BLA therapeutic drug monitoring (TDM) due to their significant PK variability. The purpose of this study was to compare clinical outcomes in patients with PSAR pneumonia (PNA) or bloodstream infection (BSI) receiving BLA with and without the guidance of TDM.

**Methods:**

Retrospective, parallel cohort study conducted at UF Shands Gainesville and UF Health Jacksonville evaluating five years of patients with PSAR PNA or BSI. TDM group was defined for routine BLA TDM compared to nonroutine BLA TDM service (non-TDM). Patients were excluded if they died before a culture result, transferred in with a positive PSAR culture, were transplant recipients, cystic fibrosis or burn injury patients. The primary outcome was a composite of presumed clinical cure defined as the absence of the following: all-cause in-hospital mortality, escalation, and/or additional antimicrobial therapy for PSAR infection after 48 hours of treatment with primary susceptible regimen due to worsening clinical status or transfer to a higher level of care.

**Results:**

Two-hundred patients were included (TDM n=95; non-TDM n=105). The overall primary composite outcome of presumed clinical cure occurred in 73% of patients (82% and 75% of the TDM and non-TDM cohorts, respectively; p=0.301). A post-hoc multivariate analysis was conducted to assess predictors of not attaining clinical cure.

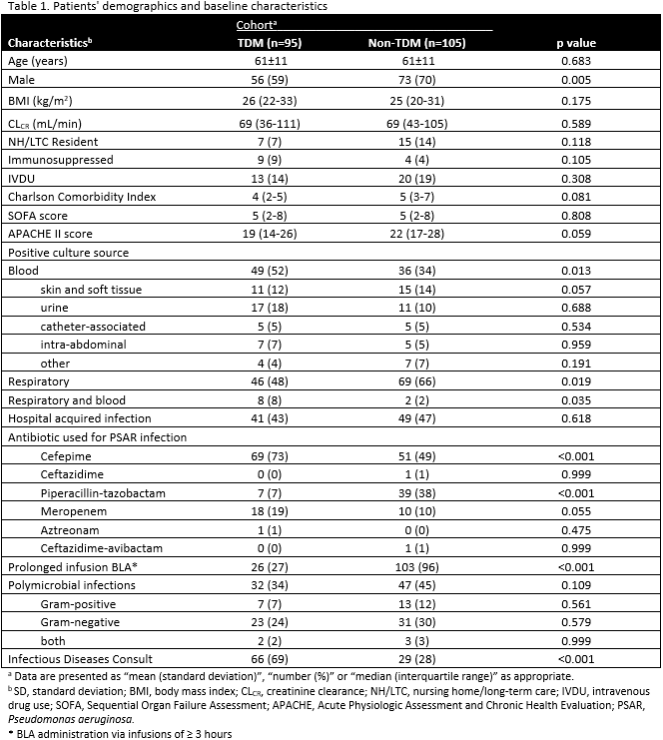

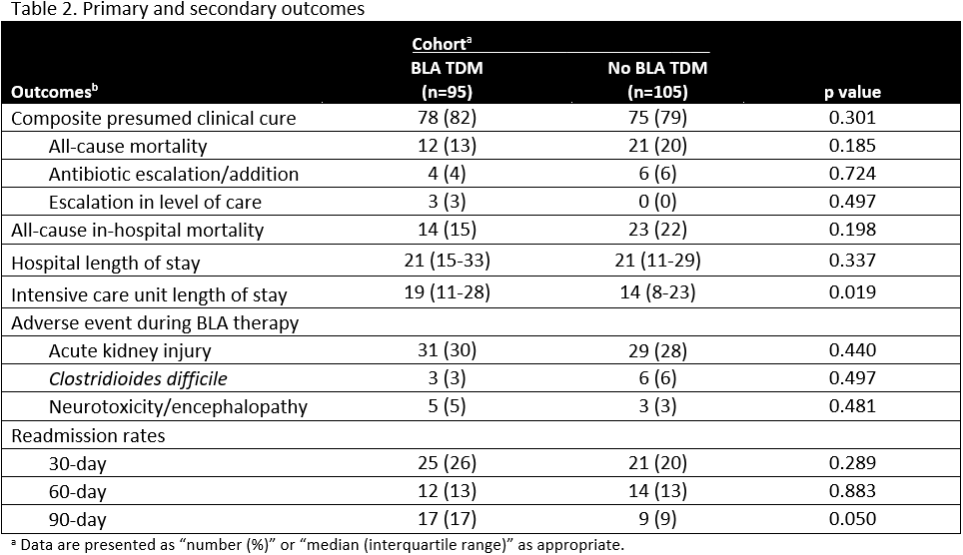

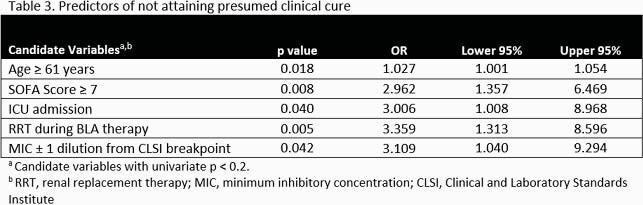

**Conclusion:**

While there was no difference in the primary composite outcome of presumed clinical cure, future studies can use these data to assess TDM patient selection and whether a bundled care approach of BLA regimens with known clinical benefit, early TDM-guided dose optimization, and continued clinical assessment improves outcomes in patients with PSAR PNA or BSI compared to use of each modality individually.

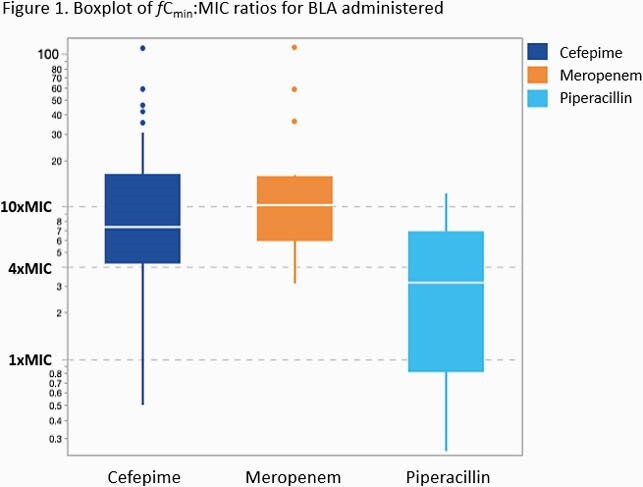

**Disclosures:**

**All Authors**: No reported disclosures

